# Therapy of human non-small-cell lung carcinoma using antibody targeting of a modified superantigen

**DOI:** 10.1054/bjoc.2001.1891

**Published:** 2001-07

**Authors:** G Forsberg, L Ohlsson, T Brodin, P Björk, P A Lando, D Shaw, P L Stern, M Dohlsten

**Affiliations:** Active Biotech Research AB, Box 724, 220 07 Lund, Sweden; Paterson Institute for Cancer Research, Manchester, UK; Department of Cell and Molecular Biology, Lund University, Sweden

## Abstract

Superantigens activate T-cells by linking the T-cell receptor to MHC class II on antigen-presenting cells, and novel reactivity can be introduced by fusing the superantigen to a targeting molecule. Thus, an antibody-targeted superantigen, which activates T cells to destroy tumour cells, might be used as cancer therapy. A suitable target is the 5T4 oncofetal antigen, which is expressed on many carcinomas. We constructed a fusion protein from a Fab of a monoclonal antibody recognizing the 5T4 antigen, and an engineered superantigen. The recombinant product 5T4FabV13-SEA_D227A_ bound the 5T4 antigen expressed on the human non-small-cell lung cancer cell line Calu-1 with a *K*_d_ of 1.2 nM while the substitution of Asp227 to Ala in the superantigen moiety reduced binding activity to MHC class II. 5T4FabV13-SEA_D227A_ tumour reactivity was demonstrated in 7/7 NSCLC samples by immunohistochemistry, while normal tissue reactivity was low to moderate. 5T4FabV13-SEA_D227A_ induced significant T-cell-dependent in vitro killing of sensitive 5T4 bearing Calu-1 cells, with maximum lysis at 10^−10^M, while the capacity to lyse MHC class II expressing cells was approximately 1000 times less effective. Immunotherapy of 5T4FabV13-SEA_D227A_ against human NSCLC was investigated in SCID mice reconstituted with human peripheral blood mononuclear cells. Mice carrying intreperitoneally growing Calu-1 cells showed significant reduction in tumour mass and number after intravenous therapy with 5T4FabV13-SEA_D227A_. Thus, 5T4FabV13-SEA_D227A_ has highly attractive properties for therapy of human NSCLC. © 2001 Cancer Research Campaign http://www.bjcancer.com


*British Journal of Cancer* (2001) **85**(1), 129–136
© 2001 Cancer Research Campaign
doi: 10.1054/ bjoc.2000.1891, available online at http://www.idealibrary.com on IDEAL®

http://www.bjcancer.com

### Therapy of human non-small-cell lung carcinoma using antibody targeting of a modified superantigen

**G Forsberg[1], L Ohlsson[1], T Brodin[1], P Björk[1], PA Lando[1], D Shaw[2], PL Stern[2] and M Dohlsten[3]**

[1]Active Biotech Research AB, Box 724, 220 07 Lund, Sweden; [2]Paterson Institute for Cancer Research, Manchester, UK;
[3]Department of Cell and Molecular Biology, Lund University, Sweden

**Summary** Superantigens activate T-cells by linking the T-cell receptor to MHC class II on antigen-presenting cells, and novel reactivity can be introduced by fusing the superantigen to a targeting molecule. Thus, an antibody-targeted superantigen, which activates T cells to destroy tumour cells, might be used as cancer therapy. A suitable target is the 5T4 oncofetal antigen, which is expressed on many carcinomas. We constructed a fusion protein from a Fab of a monoclonal antibody recognizing the 5T4 antigen, and an engineered superantigen. The recombinant product 5T4FabV13-SEA$_{D227A}$ bound the 5T4 antigen expressed on the human non-small-cell lung cancer cell line Calu-1 with a $K_d$ of 1.2 nM while the substitution of Asp227 to Ala in the superantigen moiety reduced binding activity to MHC class II. 5T4FabV13-SEA$_{D227A}$ tumour reactivity was demonstrated in 7/7 NSCLC samples by immunohistochemistry, while normal tissue reactivity was low to moderate. 5T4FabV13-SEA$_{D227A}$ induced significant T-cell-dependent in vitro killing of sensitive 5T4 bearing Calu-1 cells, with maximum lysis at $10^{-10}$ M, while the capacity to lyse MHC class II expressing cells was approximately 1000 times less effective. Immunotherapy of 5T4FabV13-SEA$_{D227A}$ against human NSCLC was investigated in SCID mice reconstituted with human peripheral blood mononuclear cells. Mice carrying intreperitoneally growing Calu-1 cells showed significant reduction in tumour mass and number after intravenous therapy with 5T4FabV13-SEA$_{D227A}$. Thus, 5T4FabV13-SEA$_{D227A}$ has highly attractive properties for therapy of human NSCLC. © 2001 Cancer Research Campaign http://www.bjcancer.com

Antibody-based therapies are currently evaluated for treatment of several severe diseases, such as cancer, viral infections and autoimmunity. Recent technological improvements have made it possible to clone and produce large amounts of intact recombinant monoclonal antibodies or antibody fragments with unique specificities (reviewed by Winter and Milstein, 1991; Dall'Acqua and Carter, 1998; Hudson, 1998). Several tumour-associated antigens have been identified and are currently being investigated as therapeutic targets for haematological and solid tumours (Riethmüller et al, 1993). non-small-cell lung cancer (NSCLC) is an aggressive solid tumour associated with a poor prognosis since surgery or chemotherapy is only beneficial in a fraction of all cases. Antibody-based therapy has been of limited success, but has been described using murine antibodies against targets such as the EGF-receptor in squamous cell carcinoma (Modjtahedi et al, 1996) or epithelial glycoprotein-2 for adenocarcinoma (Zimmermann et al, 1997). Such monoclonal antibodies probably may act by interfering in tumour cell signalling or through activation of complement and/or Fc receptor bearing cells. To potentiate the effects of monoclonal antibodies, the use of fusions with superantigens, which are of bacterial or viral origin and activate T cells by linking the T cell receptor to MHC class II on antigen presenting cells (reviewed by Johnson et al, 1992), have previously been described by us (Dohlsten et al, 1994; Brodin et al, 1998). Thereby, large amounts of cytotoxic and cytokine producing T-cells can be targeted to destroy and initiate a powerful T cell attack against tumour cells in vivo (Dohlsten et al, 1994, 1995a). Most studies of antibody targeted superantigens, e.g. staphylococcal enterotoxin A, SEA, have been for the treatment of human colorectal cancer (Dohlsten et al, 1995b). However, to decrease the reactivity of the superantigen with MHC class II-bearing cells, the Asp227Ala replacement were introduced to destroy the site having the highest affinity for MHC class II in SEA (Abrahmsén et al, 1995).

The human 5T4 antigen was discovered by looking for shared surface molecules which would reflect the functional similarities between the growth and invasive properties of trophoblast, the major interfacing cell type between mother and fetus in the placenta, and tumour cells. The murine antibody 5T4 recognizes a 72 kDa glycoprotein (Hole and Stern, 1988, 1990) found in many carcinomas, especially non-small-cell lung and breast cancer, but at low levels in normal tissues (Southall et al, 1990). 5T4 tumour-associated labelling is also a marker of prognostic significance in colorectal (Starzynska et al, 1992, 1994, Mulder et al, 1997) and gastric carcinoma (Starzynska et al, 1998). The cDNA encoding human 5T4 predicts a heavily glycosylated membrane-bound protein with regions containing leucine rich repeats, which probably contribute directly to protein–protein interactions (Myers et al, 1994). Overexpression of 5T4 antigen alters cell adhesion, shape and motility in vitro (Carsberg et al, 1995, 1996). Minimal residual disease is likely to be 5T4 positive and is thus a potential candidate for superantigen mediated therapy. Here, the potential for a fusion protein between 5T4 and SEA$_{D227A}$ was investigated for therapy of NSCLC.

*Received 8 June 2000*
*Received 8 November 2000*
*Accepted 17 November 2000*

*Correspondence to: G Forsberg*

### MATERIALS AND METHODS

### Cells

The 5T4 hybridoma was grown and the mAb purified as previously described (Hole and Stern, 1990). The human leukaemia cell line K562, colon adenocarcinoma WiDR, HT29, NSCLC Calu-1, ME 180 and the B cell lymphoblastoid Raji were obtained from American Type Culture Collection (Rockville, MD). All cells were mycoplasma free and maintained under standard conditions.

Peripheral blood mononuclear cells, PBMC, were from heparinized blood from normal donors at the University Hospital of Lund. The cells were isolated by density centrifugation over Ficoll-Paque cushion and incubated in complete R-medium (RPMI-1640 supplemented with 10% fetal calf serum (Gibco BRL, Life Technologies, UK), 1 mM glutamine, 10 mM Hepes buffer, 1 mM sodium pyruvate (HyClone Europe, UK), 50 µM 2-mercaptoethanol (ICN Biomedicals INC. Costa Mesa CA), 0.1 M $NaHCO_3$ (Seromed Biochrome), 0.1 mg $ml^{-1}$ gentamycine (Biological Industries, Kibbutz Beit Haemek, Israel). SEA activated T cell lines were produced by activation of PBMC using $2 \times 10^6$ cells $ml^{-1}$ with mitomycin C-treated BSM cells preincubated with 100 ng $ml^{-1}$ SEA in medium with 10% FCS (Dohlsten et al, 1991). The T cell lines were restimulated biweekly with 20 U $ml^{-1}$ IL-2, weekly with mitomycin C treated SEA coated BSM cells (Van De Griend et al, 1984) and cultivated for 4–12 weeks before being used in the assay. The viability of the effector cells, as determined by trypan blue exclusion, exceeded 50%. Flow cytometric analysis and sorting were performed according to standard setting on a $FACStar^{Plus}$ (Becton Dickinson, Mountain View, CA, USA).

### Cloning, expression and purification of Fab-SEA fusion proteins

The fusion proteins were produced at Pharmacia & Upjohn (Stockholm, Sweden) essentially as described (Dohlsten et al, 1994; Abrahmsén et al, 1995; Forsberg et al, 1997). The Fv-encoding portions of 5T4 were cloned from the 5T4 hybridoma and fused to sequences coding for the constant regions of the murine IgG1/k antibody C242 lacking the interchain disulphide bond. A region coding for $SEA_{D227A}$ was connected to the C-terminus of the heavy chain (Figure 1A) via a Gly-Gly-Pro linker. The products were expressed and secreted in the *E. coli* K-12 strain UL 635 (*xyl*-7, *ara*-14, $T4^R$, Δ*omp*T) (Abrahmsén et al, 1995) using a plasmid with a lacUV5-promoter. After fermentation, clarified growth medium was applied to a Protein G Sepharose column (Pharmacia Biotech, Uppsala, Sweden) and bound protein eluted with 0.1 M acetic acid, 0.05% Tween 80. Full-length product was separated from a degraded variant lacking the superantigen moiety, 5T4FabV13, on an SP Sepharose HP column (Pharmacia Biotech) using a linear gradient from 60 to 350 mM sodium acetate (Forsberg et al, 1997). SDS-PAGE and chromatographic techniques indicate that the purity of the product was at least 95%.

### Assays

To measure lymphocyte proliferation by incorporation of [$^3$H]-thymidine, $2 \times 10^5$ PBMC were incubated at 37˚C in 200 µl complete R-medium with titrating amounts of Fab-SEA proteins for 72 h, as described (Dohlsten et al, 1988). Tumour cell growth-inhibition assay were performed using $5 \times 10^3$ tumour cells well in 96-well flat-bottomed microtitre wells (Nunc, Roskilde, Denmark) in complete R-medium and titrating dilutions of supernatants from PBMC incubated for 72 h with $10^{-9}$ M of Fab-SEA fusion proteins in a total volume of 200 µl. Tumour cells were then incubated for 72 h and the viable fraction of cells determined using the MTT-assay (Van de Loosdrecht et al, 1991). The production of IL-2, IFN-γ and TNF-α in $2 \times 10^6$ PBMC $ml^{-1}$ incubated at 37˚C with titrating amounts of Fab-SEA proteins, were measured in culture supernatants from plates in 2 ml R-medium with specific ELISA reagents (Genzyme Corporation, Cambridge, MA) as recommended by the supplier.

To study cytotoxicity, K562, Calu-1 or Raji cells, labelled with $(Na)_2{}^{51}CrO_4$ (Amersham Solna, Sweden) were used in a standard 4 h chromium release assay (Dohlsten et al, 1994). As effector cells, an SEA reactive T cell line, prepared as described above, was used. This cell line contains more than 99% CD3-positive cells (Hedlund et al, 1990). The specific cytotoxicity was calculated using the average counts/min (cpm) in the formula: specific cytotoxicity = (experimental cpm – spontaneous release cpm)/(total release cpm – spontaneous release cpm).

### Binding assays

Affinities to the 5T4 antigen were measured similarly to Forsberg et al (1997). 5T4FabV13-$SEA_{D227A}$ and 5T4FabV13 respectively were labelled with $Na^{125}I$ (NEN, Boston, MA) to obtain a specific activity of 10 to 40 µCi $µg^{-1}$ protein and an iodine to protein ratio of ≤ 2:1. Serially diluted $^{125}$I-labelled 5T4FabV13-$SEA_{D227A}$ or 5T4FabV13 in triplicate was incubated with Calu-1 or ME 180 cells for 2 h at room temperature. After washing, cell-bound radioactivity was determined. The dissociation constant, $K_d$, and number of binding sites, $N$, at saturation were calculated (Scatchard, 1949) after subtraction of non-specific binding. To determine affinities to MHC class II, plasma membranes were prepared from Raji cells (Massague, 1987). Approximately $10^8$ frozen cells were homogenized in 10 mM Tris-HCl, 1 mM EDTA, 1 mM PMSF, pH 7, containing 0.25 M sucrose with 20 strokes in a pre-cooled Dounce homogenizer and centrifuged 10 min at 6000 rpm. The pellet was resuspended in that buffer with sucrose and centrifuged. The combined supernatants were layered on top of a cushion containing 37% w/v sucrose in buffer and centrifuged at 105 000 $\boldsymbol{g} \times 60$ min. The membrane layer was removed, diluted 4 times with buffer and centrifuged at 30 000 $\boldsymbol{g} \times 30$ min. The pellet was resuspended in 1 ml buffer and stored at –70 ˚C. Plasma membranes were immobilised as described by Vater et al (1995). Aliquots from fractionated plasma membranes were diluted in 5 mM $NaN_3$ and 1 mM PMSF and distributed on a 96-well Immulon 2 ELISA plate (Dynatech Labs). The plates were dried, unbound sites blocked for 1 h and washed twice in Tris-buffered saline containing 3% defatted milk. Serially diluted biotinylated SEA (long-arm NHS-biotin reagent, Vector Labs) was incubated with 5T4FabV13-$SEA_{D227A}$, SEA or C242Fab-SEA in blocking buffer for 5 min and then membranes for 2 h at room temperature. Detection was carried out using the Vectastain® ABC Kit (BioRad).

### Immunohistochemistry

7 histologically characterized samples of NSCLC (5 adenocarcinoma and 2 squamous cell carcinoma) were from Dr Jayant Shetye

 *© 2001 Cancer Research Campaign*

(Department of Pathology, Karolinska Hospital). Normal tissues (see Table 1) and breast tumours were provided by the Department of Surgery, Lund University Hospital. Acetone fixed cryosections of the above tissues were labelled with 5T4FabV13SEA$_{D227A}$ followed by biotinylated rabbit anti-SEA (in house production). After incubation in streptavidin-biotin/horseradish peroxidase (Dako, Copenhagen) the sections were developed in diaminobenzidine (Saveen AB, Malmö), counterstained in methyl green and mounted. As reference to reaction seen in vessels anti human CD31 antibody was used (Dako, Copenhagen). As negative controls irrelevant FabSEA$_{D227A}$ and mouse monoclonal IgG1 were used.

### In vivo induction of cytokines

All animal were kept under pathogen-free conditions and the experiments carried out using approved ethical protocols. C57/B16 mice got 4 daily intravenous injections of 30 υg control Fab-SEA or 5T4FabV13-SEA$_{D227A}$ in PBS or buffer alone. Blood samples were taken by caval vein puncture at various time points. All groups contained pooled sera from 3 animals. The levels of IL-2, IL-6, TNF-α and IFN-γ were measured as above.

### Therapy in SCID mice

Severe Combined Immunodeficient (SCID) female mice (C.B-17, Bommice, Ry, Denmark) were injected intraperitoneally with $3 \times 10^6$ Calu-1 cells in vehicle (0.2 ml PBS-1%Balb/c mouse serum) and 5 days later I.P. with $3 \times 10^6$ PBMC in 0.2 ml vehicle. 1 to 2 h after injection of PBMC all mice were injected intravenously with 5T4FabV13-SEA$_{D227A}$ or the non-binding control C215Fab-SEA$_{D227A}$ (Hansson et al, 1997) in 0.2 ml vehicle or vehicle alone. 2 additional intravenous injections of the respective test substance were given with 3 day intervals unless otherwise specified. The mice were sacrificed between day 30 to 40, by cervical dislocation and the number of tumours and the tumour mass determined. Tumours of less than 5 mg were estimated as 2 mg, tumours with a mass of more than 5 mg and less than 10 mg as 7 mg and tumours larger than 10 mg with the actual weight. All tumours larger than 1 mg were counted. Each treatment cohort contained 5 to 7 mice to permit comparison to other treatment cohorts treated simultaneously with the same batch of effector cells. Statistical significance was determined by the Mann–Whitney U test. For histochemical analysis, 6 mm cryosections were analysed as above.

### RESULTS

### *E. coli* production of the fusion protein

The fusion protein 5T4FabV13-SEA$_{D227A}$ (Figure 1A) was produced as a secreted product in *E. coli*. The product was expressed as bicistronic construct with SEA$_{D227A}$ fused to the C-terminus of the Fab heavy chain. The production level was increased 15-fold by replacing 7 amino acid residues in the framework of 5T4Fab, yielding 5T4FabV13 (Forsberg et al, 1997). The yield in the *E. coli* growth medium of 5T4FabV13-SEA$_{D227A}$ was in the order of 0.5 g $l^{-1}$. To decrease the MHC class II binding, and subsequently in vivo toxicity, the replacement Asp227Ala was introduced in SEA, yielding SEA$_{D227A}$ (Abrahmsén et al, 1995). The full-length product was recovered in a 2-step purification procedure. The purity of the product was at least 95% as determined by SDS-PAGE and chromatographic techniques. In addition to the full-length product, a truncated variant corresponding to 5T4V13Fab, was recovered in the second purification step.

### Tissue reactivity of 5T4FabSEA$_{D227A}$

The expression of 5T4 antigen in tumour and normal tissue was investigated with immunohistochemistry. 5T4FabV13-SEA$_{D227A}$

**A**

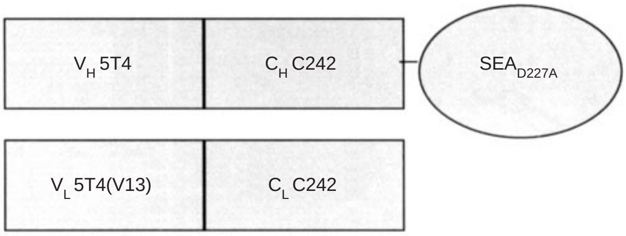

**B**

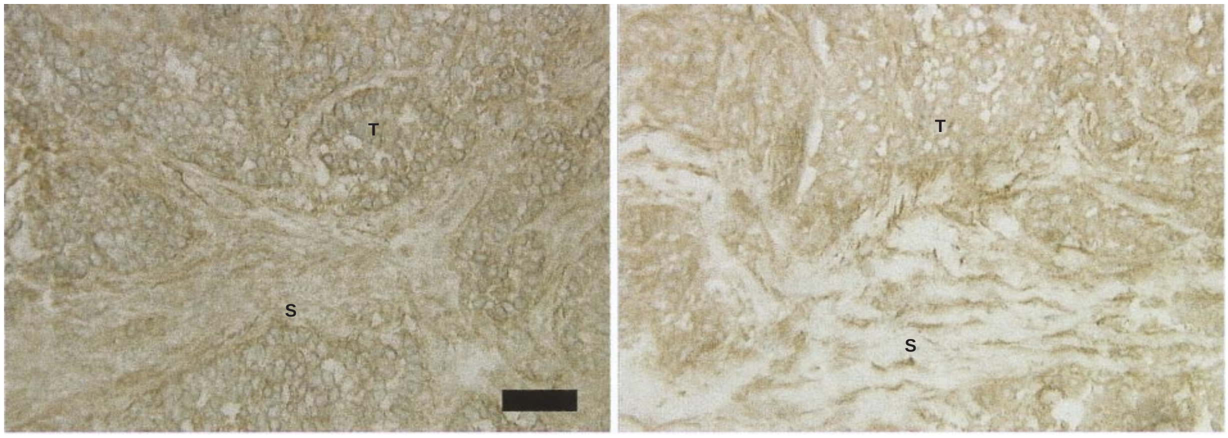

NSCLC    Breast cancer

**Figure 1** (**A**) Schematic representation of 5T4FabV13-SEA$_{D227A}$. The product consists of a modified variant of the variable regions from 5T4 antibody (Forsberg et al, 1997) connected to the CL and CH1 domains of the antibody C242 (Dohlsten et al, 1994). The superantigen SEA$_{D227A}$ is fused to the C-terminus of CH1. (**B**) Immunohistochemical analysis of the 5T4-antigen expression in NSCLC tumour tissue and breast cancer tissue. Strong expression is observed on both the tumour cells (T) as well as in the stroma (S)

*© 2001 Cancer Research Campaign*

**Table 1** Summary of the immunohistochemical analysis of NSCLC, breast cancer and normal tissue. The tissue-reactivities were scored semiquantitatively according to the intensity of the staining. A + equals weak reaction, ++ moderate reaction and +++ strong reaction

| Tissue | Reactivity | Comment |
|---|---|---|
| NSCLC (*n* = 7) | ++ –+++ | Homogenous staining in 7/7 samples, 5 adenocarc. and 2 squamous carc. |
| Breast ca. (*n* = 6) | ++ –+++ | Homogenous staining in 6/6 samples. |
| CNS (*n* = 1) | neg. | |
| Skin (*n* = 2) | neg. | |
| Myocardium (*n* = 4) | + | Reaction in the luminal outline of a subpopulation of muscular vessels in 2/4 samples. |
| Adrenal (*n* = 2) | neg. | |
| Kidney (*n* = 4) | + –++ | Weak-moderate diffuse reaction in glomeruli and parietal layer outlining Bowman's capsule (2/4). Weak focal reaction outlining lumen in occasional muscular vessels (2/4). |
| Lung (*n* = 4) | + –++ | Weak luminal outline in occasional vessels (2/4). Moderate reaction in a basal epithelial cellular or matrix component associated with the bronchial epithelium. |
| Liver (*n* = 4) | + | Occasional staining of the sinusoidal outline close to the central vein (1/4). |
| Pancreas (*n* = 2) | + –++ | Weak-moderate reaction in occasional pancreatic ducts and scarce stroma structures. Weak focal reaction outlining lumen in occasional muscular vessels. |
| Gastro-intestinal tract (stomach *n* = 2, small intestine *n* = 2 and large intestine *n* = 4) | ++ | Reaction in some cell type or extracellular component of the epithelial basal lamina or the lamina propria in parts of the surface epithelium |
| Pharynx (*n* = 2) | + –++ | Reaction in squamous epithelium (most prominent in basal layer). |
| Thyroid (*n* = 2) | + | Reaction associated with follicular epithelial cells. Focal reaction outlining lumen of occasional muscular vessels. |
| Spleen (*n* = 2) | + | Focal reaction outlining lumen of occasional muscular vessels. |

tumour reactivity was demonstrated in 7/7 cases of NSCLC, including 5 adenocarcinomas and 2 squamous cell carcinomas (Table 1). Moderate staining was seen in 4 of the adenocarcinomas and 1 squamous cell carcinoma and moderate to strong in the remaining. In a group of 6 breast carcinomas, moderate reactions were seen in 5 of them and a moderate–strong in the sixth. Reaction was not only confined to the tumour cells since all examined tumours (both NSCLC and breast carcinomas) also showed stromal reactivity (Figure 1B). In some cases the stromal reaction dominated over the tumour cell reaction. 5T4FabV13-SEA$_{D227A}$ reactivity was also assessed in some normal tissues (Table 1) and was found to be similar to that seen with the mAb (Southall et al, 1990). This reactivity was not observed with an irrelevant FabSEA$_{D277A}$ protein. The normal tissue reactivity is presented in Table 1. The most quantitatively dominating normal tissue reactivity was found in some cell type or extra-cellular component found in association with the basal membrane or the lamina propria of the alimentary tract. Focally weak reaction was seen in the luminal outline of a minority (less than 10%) of muscular blood vessels in different normal tissues. This reaction showed individual variation since only 2/4 colon samples, 2/4 lung and 2/4 myocardial samples demonstrates it.

In kidney 2/4 showed weak diffuse, possibly intracellular reaction in glomeruli and outlining of the parietal layer of Bowman's capsule while the other 2 showed weak–moderate reaction in these structures. In the liver 1/3 samples showed a weak staining outlining sinusoids proximal to the central vein. Weak–moderate reaction was also found in duct epithelium of pancreas and in squamous epithelium of pharynx and weak reaction was found in the epidermal layer of the skin and in association with the follicular epithelium of the thyroid gland.

### Binding affinity of 5T4FabV13-SEA$_{D227A}$ to the 5T4 antigen and MHC class II

Several cell lines were investigated for 5T4 antigen expression using flow cytometry and the strongest 5T4 FACS positive NSCLC line, Calu-1, was used to measure the affinity of the fusion protein to the 5T4 antigen. In FACS analysis, the fusion protein 5T4FabV13-SEA$_{D227A}$ bound to the cells in a dose-dependent manner with maximum binding at $10^{-8}$ M (data not shown). Radioiodinated 5T4FabV13-SEA$_{D227A}$ and 5T4FabV13 were used for the antigen-binding assays. Figure 2A shows that both reagents have nM affinity (mean $K_D$ of $1.2 \times 10^{-9}$ M or $2.3 \times 10^{-9}$ M respectively) with an antigen density of approximately $3 \times 10^5$ molecules per cell. Binding of 5T4FabV13-SEA$_{D227A}$ was also measured on ME 180 cells. Here, the affinity was slightly higher, $0.7 \times 10^{-9}$ M, while the number of binding sites were approximately $1.3 \times 10^5$ molecules per cell (data not shown).

The binding to MHC class II expressed on Raji cells was then investigated. In accordance with previous findings (Dohlsten et al, 1994), Fab-SEA was a much weaker competitor for binding to MHC class II molecules than SEA. SEA and Fab-SEA showed $IC_{50}$ values of approximately 21 and 360 nM respectively in a competitive assay with biotinylated SEA to coated Raji cell plasma membranes. 5T4FabV13-SEA$_{D227A}$ did not show any displacement in the concentration range used (≤ 20 µM) and thus has an affinity of less than 10 µM (Figure 2B).

### Cytotoxicity of 5T4FabV13-SEA$_{D227A}$ to NSCLC and MHC class II-expressing cells

To investigate T-cell mediated cytotoxicity on tumour cells induced by the fusion protein in vitro, 5T4FabV13-SEA$_{D227A}$ was

 © 2001 Cancer Research Campaign

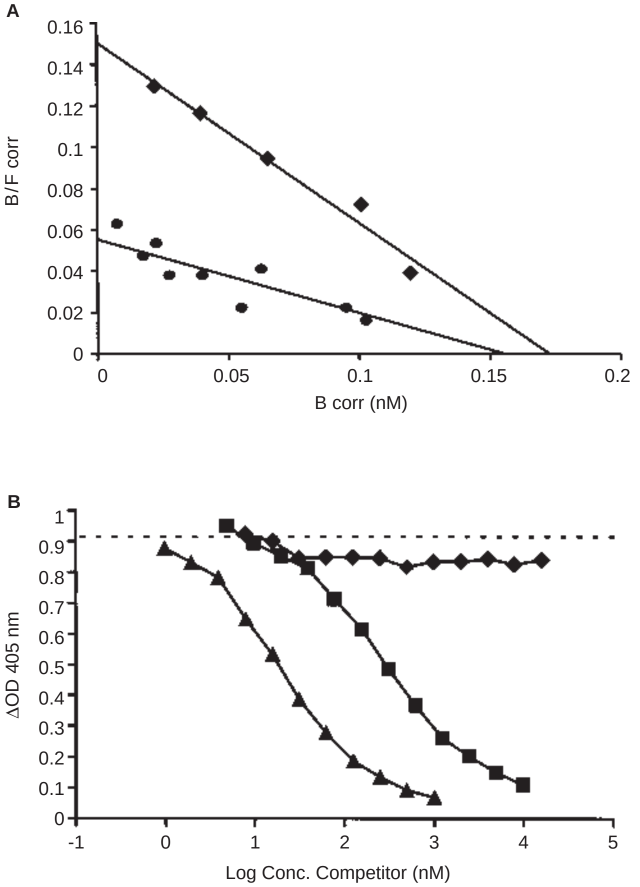

**Figure 2** (**A**) Representative Scatchard plot and saturation curve for binding of $^{125}$I-5T4FabV13-SEA$_{D227A}$ (◆) and $^{125}$I-5T4FabV13 (●) to Calu-1 cells. Values are corrected for non-specific binding. The calculated $K_d$ in this experiment were 1.2 and 2.3 nM respectively with 300 000 sites/cell. Each point represents the mean of triplicates. (**B**) Binding of 10 nM biotinylated SEA to immobilized MHC class II positive Raji cell plasma membranes in the presence of SEA (▲), C242Fab-SEA (Dohlsten et al, 1995b) (■) or 5T4FabV13-SEA$_{D227A}$ (◆) as competitors. Each point represents the mean of duplicates

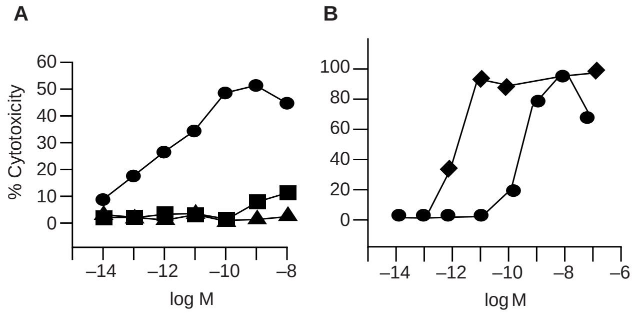

**Figure 3** Induced cytotoxicity using 5T4FabV13-SEA$_{D227A}$ on (**A**) chromium-labelled 5T4 expressing Calu-1 cells and (**B**) chromium-labelled MHC class II-expressing Raji cells. The symbol ● represents 5T4FabV13-SEA$_{D227A}$, ■ control Fab-SEA$_{D227A}$, ▲ 5T4FabV13-SEA$_{D227A}$ in the absence of T cells and ◆ control Fab-SEA. The effects were mediated by fusion proteins and human activated T cells. The cells were incubated for 4 h, then the released chromium was measured and the % cytotoxicity determined. 5T4FabV13-SEA$_{D227A}$ was only active in the presence of T cells and at least 100-fold more potent against Calu-1 cells

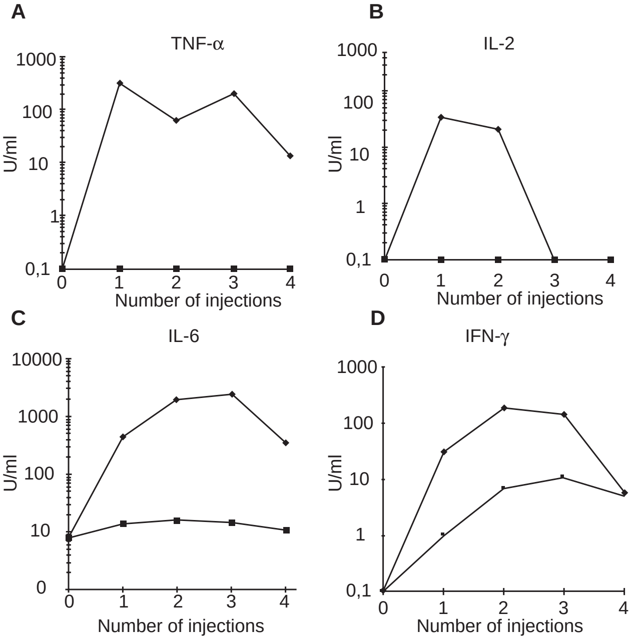

**Figure 4** Serum cytokine levels after injection of C215Fab-SEA (◆) (Dohlsten et al, 1994) and 5T4FabV13-SEA$_{D227A}$ (■) in C57/B16 mice. The use of the mutated superantigen significantly reduced the systemic release of (**A**) TNF-α, (**B**) IL-2, (**C**) IL-6 and (**D**) IFN-γ. A 10- to 100-fold reduction in the serum levels of IL-2 and IFN-γ and a 100 to 1000 reduced amount of TNF-α and IL-6 was recorded in animals treated with SEA$_{D227A}$ containing fusion protein mixed with chromium labelled Calu-1 cells and human SEA reactive T cells (Dohlsten et al, 1991). 5T4FabV13-SEA$_{D227A}$ mediated a specific T cell killing of Calu-1 cells (Figure 3A) and was $10^5$ times more effective than control Fab-SEA$_{D227A}$, as determined by the EC$_{50}$ value. The EC$_{50}$ was approximately $10^{-12}$ M for 5T4FabV13-SEA$_{D227A}$ and no cytotoxicity was observed in the absence of T cells. 5T4FabV13-SEA$_{D227A}$ was also tested for the ability to mediate MHC class II-dependent superantigen-mediated cytotoxicity against chromium-labelled Raji cells (Figure 3B). 5T4FabV13-SEA$_{D227A}$ had about 100 times reduced MHC class II-dependent cytotoxicity compared to control Fab-SEA, as judged by the EC$_{50}$ value. This reflects the lowered affinity to MHC class II by the D227A substitution in SEa. In this assay, the EC$_{50}$ value of 5T4FabV13-SEA$_{D227A}$ was approximately $10^{-10}$ M.5T4FabV13-SEA$_{D227A}$ had similar activity as the control Fab-SEA$_{D227A}$.

### In vivo immune activation of 5T4FabV13-SEA$_{D227A}$

In order to quantify the systemic immune response by Fab-SEA$_{D227A}$ relative to Fab-SEA proteins, we analysed the serum levels of a panel of cytokines in C57/B16 mice injected with the same dose Fab-SEA and 5T4FabV13-SEA$_{D227A}$. There is a correlation between the systemic cytokine levels and systemic toxicity for Fab-SEA constructs in both mice and humans (Dohlsten et al, 1995b; Alpaugh et al, 1998). Repeated injections of Fab-SEA resulted in strong production of IL-2, IFN-γ, TNF-α and IL-6 (Figure 4). Drastic reduction in the systemic levels of all tested cytokines was seen when comparing the effects of Fab-SEA with 5T4FabV13-SEA$_{D227A}$. A 10-to 100-fold reduction in the serum levels of IL-2 and IFN-γ and a 100 to 1000 reduced amount of

© 2001 Cancer Research Campaign

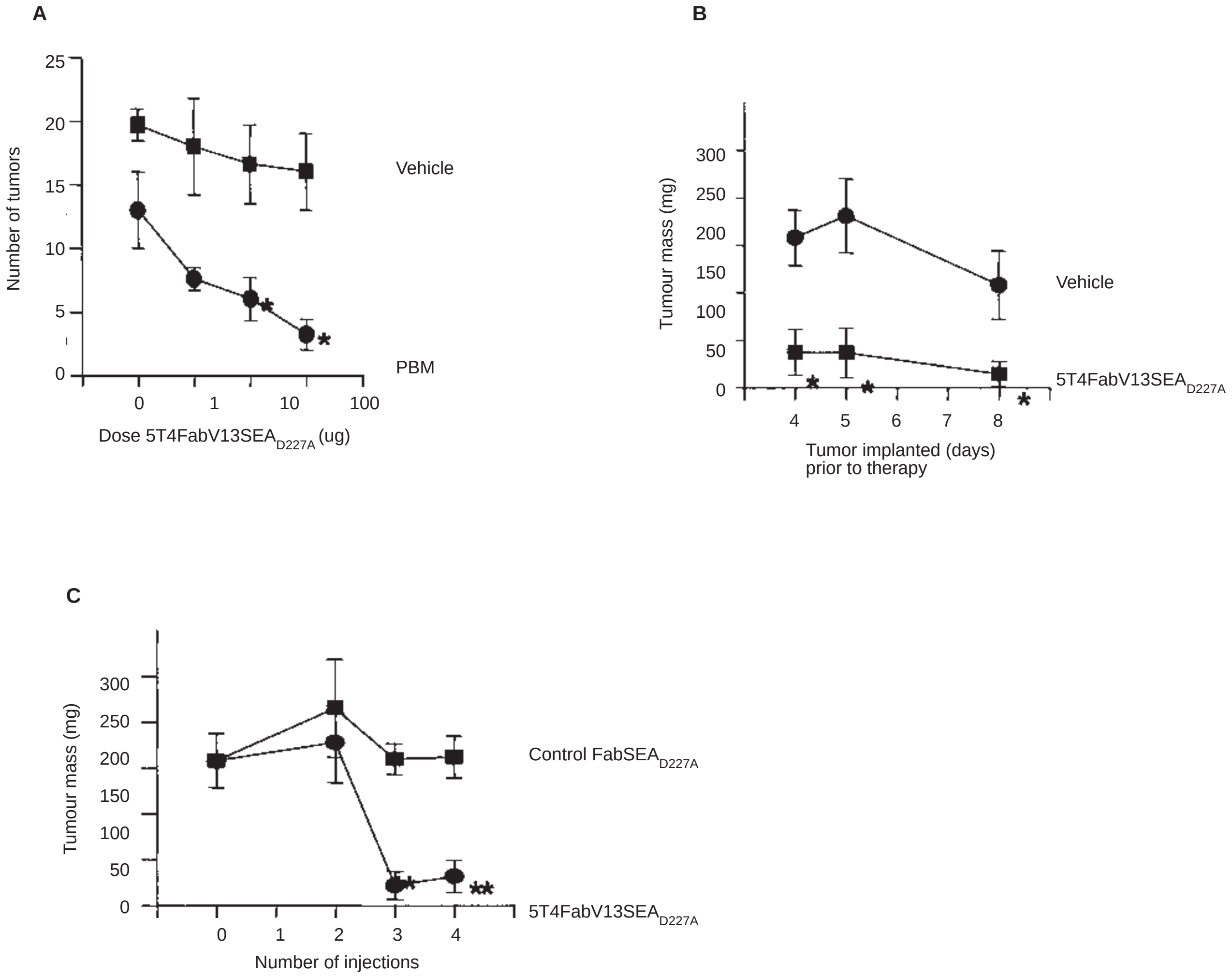

**Figure 5** Therapy of SCID-mice carrying human Calu-1 tumours. (**A**) Dose titration, (**B**) effects of therapy on day 4, 5 and 8 tumours and (**C**) increased effects are observed using repeated injections of 5T4FabV13SEA$_{D227A}$. 5T4FabV13-SEA$_{D227A}$ was active even on day 8 tumours, but only in the presence of PBM. Best effects were observed after repeated injections

TNF-α and IL-6 was recorded in animals treated with 5T4FabV13-SEA$_{D227A}$ (Figure 4). In fact, the levels of IL-2 and TNF-α were below the level of detection for the assay using the superantigen analogue. Thus, the replacement in the MHC class II-binding region in SEA results in a drastic reduction in the systemic levels of cytokines in the murine system.

### Therapy of human NSCLC with 5T4FabV13-SEA$_{D227A}$ in humanized SCID mice

To investigate 5T4FabV13-SEA$_{D227A}$ therapy against human NSCLC cells, a SCID-mouse model was developed. Calu-1 cells were tested for intraperitoneally growth in the mice (data not shown) and kinetic analysis showed an increased tumour growth during the first 30 days. All tumours were found to be 5T4 antigen positive using immunohistochemical staining 9, 10 and 25 days after transplantation. Using systemic intravenous treatment, 5T4FabV13-SEA$_{D227A}$ was given 3 times with 3 day intervals of mice grafted with human PBMC, a strong suppression in Calu-1 tumour mass and number was obtained (Figure 5). More than 85% reduction in the number of tumours and more than 95% reduction in tumour mass, calculated as described in Materials and methods, were observed. No significant therapy was observed with a control fusion protein not binding to the Calu-1 tumour, indicating that the therapy involves specific targeting to 5T4 positive tumour cells. Significant tumour therapy was only observed when treating with 5T4FabV13-SEA$_{D227A}$ in the presence of PBMC (Figure 5). The 5T4FabV13-SEA$_{D227A}$ therapy was dose-dependent requiring 10 μg/injection or more for a significant effect. Depending on the T-cell donor however, significant therapy can sometimes be seen with 1 μg/injection of 5T4FabV13-SEA$_{D227A}$ (data not shown). 3 or more injections of 5T4FabV13-SEA$_{D227A}$ were required for optimal tumour therapy in the humanised SCID mice (Figure 5). Significant therapy of Calu-1 growth was obtained against 4, 5- and 8-day-old established tumours (Figure 5). A more than 85% reduction of tumour weight was observed on 8-day-old established tumours.

Thus, significant dose-dependent tumour therapy against established NSCLC tumours growing in humanized SCID mice was obtained with 5T4FabV13-SEA$_{D227A}$.

 *© 2001 Cancer Research Campaign*

### DISCUSSION

NSCLC is associated with a poor prognosis since the beneficial effects of the available therapies are limited. Current therapeutic protocols include surgery and chemotherapy, but despite recent improvements most advanced stage patients die of the disease. However, it has clearly been shown that human non-small-cell lung cancer tissue contain tumour-infiltrating lymphocytes that upon activation releases tumoricidal cytokines (Ortegel et al, 2000). Thus, the fusion protein 5T4FabV13-SEA$_{D227A}$ represents a novel and attractive approach for therapy of NSCLC. The 5T4FabV13 has a high affinity for the antigen and can therefore be used for efficient targeting of superantigens to the tumour tissue. The SEA variant used has very potent T cell activating as well as cell killing properties and it has been modified to reduce systemic toxicity. Fusing the superantigen and 5T4FabV13 has not significantly altered their individual properties and the recombinant product can be produced at very high levels in *E. coli*, which is not always the case of recombinant antibody fragments (reviewed by Hudson, 1998). The favourable reactivity of the 5T4 antigen in all tested NSCLC and breast carcinomas in combination with the low normal tissue reactivity suggests that these types of cancer cells constitute good targets for the fusion protein. Also, the tumour stroma contained large amounts of the 5T4 antigen (Figure 1) and may therefore be an additional target for the fusion protein. Whether the binding of 5T4FabV13-SEA$_{D227A}$ to stromal cells contributes in the eradication of solid tumours remains to be studied. Most of the normal tissue reactivity found was found to be weak and focal/-diffuse. The reaction associated to the gastro-intestinal tract is the most prominent. However, the nature of this reactivity, whether it is cell-bound, cell surface associated or extracellular can not be concluded from light microscopy analysis and thus it is not possible to make predictions of 5T4FabV13-SEA$_{D227A}$ targeting to these structures in vivo. This is also the case for the reaction seen in association to lung bronchial epithelium. In a previous clinical phase I study, no obvious organ-related side effects were seen in cancer patients using C242FabSEA (Alpaugh et al, 1998) which binds strongly to MHC class II as well as to normal colon tissue.

Treatment of certain neoplastic disease with monoclonal antibodies is effective. Very encouraging data has been presented for B-cell malignancies (McLaughlin et al, 1998), colorectal cancer (Riethmüller et al, 1998) as well as Her-2 positive breast cancer (Goldenberg, 1999). Traditionally murine antibodies were used, but more recently human or humanized antibodies have shown to have advantages. There is an intense focus on other antibodies in the preclinical or early clinical phase, but there are also activities ongoing to further potentiate the successful antibodies using radioisotopes, cytotoxic fusion partners or by making the antibodies bispecific. Targeting of superantigens has previously been described for colon cancer therapy (Dohlsten et al, 1994, 1995b). This therapy leads to infiltration of T-cells in the tumour tissue (Dohlsten et al, 1995a, Litton et al, 1996). Superantigen therapy stimulates both CD4+ and CD8+ T-cells (Dohlsten et al, 1995a) that have potent cytotoxic properties to directly kill target cells, but secondary effects such as cytokine secretion and recruitment of other effector cells may be of even higher importance for successful therapy. These secondary events are important to kill sub-populations of cancer cells not expressing the antigen. Targeting of wild-type SEA has been investigated in phase I clinical studies for colon cancer therapy (Alpaugh et al, 1998). Preclinical data also suggest that these already powerful molecules can be further potentiated by the simultaneous targeting of IL-2 to the tumour tissue (Rosendahl et al, 1999; Soegaard et al, 1999). Studies using bispecific antibodies and staphylococcal enterotoxin B (Rice et al, 1999), show that this combination induce anti-tumour immunity and since 5T4FabV13-SEA$_{D227A}$ uses similar immunological principles for therapy, it is tempting to speculate that also here a T-cell memory is induced.

The structure and function properties of SEA have been well characterized (Abrahmsén et al, 1995; Hudson et al, 1995; Schad et al, 1995). To reduce the systemic toxicity, a major drawback in the use of wild-type SEA (Alphaug et al, 1998), a substitution of Asp 227 has been made. This residue co-ordinates a $Zn^{2+}$ ion to form a high affinity binding site to the β-chain of MHC class II. However, binding to MHC class II is important to obtain inflammatory cytokines and to activate T-cells. Therefore, the second MHC class II binding site surrounding Phe 47 was not altered. The biological consequences of this replacement is that the soluble fusion protein is less potent in activating resting T cells and stimulating cytokine production, due to a lower MHC class II affinity. Although the mouse is much less sensitive to SEA-induced immune activation, our data clearly demonstrates the relative potency difference in the mutated superantigen construct. This difference has been confirmed in other species such as the rabbit and monkey (data not shown), and supporting data are being obtained in humans. However, the fusion protein is equally very active when presented on a cell surface via the 5T4-antigen. Therefore, it is anticipated that the product will be very potent locally, e.g. in the tumour, while being less potent in systemic T-cell activation. Also, targeting to tissues such as the spleen is less pronounced with the mutated superantigen (Hansson et al, 1997).

One important attribute of SEA therapy is that it does not depend on the MHC-restricted T-cell recognition of peptide antigens where evasion of natural or induced CTLs may occur by down-regulation of HLA class I expression (Keating et al, 1995; Gariddo et al, 1997). There are now several well-established examples of such immune escape in the natural history of neoplasia (Bontkes et al, 1998, 1999) and there is little doubt that this presents a major problem for cancer vaccine therapies aimed at inducing CTL responses.

In conclusion, 5T4FabV13-SEA$_{D227A}$ combines excellent tumour cell-binding properties with the powerful cytotoxic properties carried by the superantigen and represents a novel type of therapy against non-small-cell lung cancer. The 5T4FabV13-SEA$_{D227A}$ is currently investigated in a phase I clinical study in NSCLC patients.

### ACKNOWLEDGEMENTS

We thank Anna Rosén, Julia Selmani, Kristina Behm, Ulrika Petterson, Anneli Nilsson, Maria Lassen, Ingegerd Andersson, Charlotte Nordenberg and Christine Valfridsson for skillful technical assistance. We are grateful to Ebba Florin-Robertsson and Mattias Widegren for the production of the fusion protein.

### REFERENCES

Abrahmsén L, Dohlsten M, Segren S, Björk P, Jonsson E and Kalland T (1995) Characterization of two distinct MHC class II binding sites in the superantigen staphylococcal enterotoxin A. *EMBO J* **14**: 2978–2986

Alpaugh RK, Weiner LM, Persson R and Persson B (1998) Overview of clinical trials employing antibody-targeted superantigens. *Adv Drug Deliv Rev* **31**: 143–152

© 2001 Cancer Research Campaign

Bontkes HJ, van Duin M, de Gruijl TD, Duggan-Keen MF, Walboomers JM, Stukart MJ, Verheijen RH, Helmerhorst TJ, Meijer CJ, Scheper RJ, Stevens FR, Dyer PA, Sinnott P and Stern PL (1998) HPV 16 infection and progression of cervical intra-epithelial neoplasia: analysis of HLA polymorphism and HPV 16 E6 sequence variants. *Int J Cancer* **78**: 166–171

Bontkes HJ, de Gruijl TD, Bijl A, Verheijen RH, Meijer CJ, Scheper RJ, Stern PL, Burns JE, Maitland NJ and Walboomers JM (1999) Human papillomavirus type 16 E2-specific T-helper lymphocyte responses in patients with cervical intraepithelial neoplasia. *J Gen Virol* **80**: 2453–2459

Brodin TN, Persson R, Soegaard M, Ohlsson L, d'Argy R, Olsson J, Molander A, Antonsson P, Gunnarsson P-O, Kalland T and Dohlsten M (1998) Man-made superantigens: Tumour selective agents for T-cell-based therapy. *Adv Drug Deliv Rev* **31**: 131–142

Carsberg CJ, Myers KA, Evans GS, Allen TD and Stern PL (1995) Metastasis-associated 5T4 oncofoetal antigen is concentrated at microvillus projections of the plasma membrane. *J Cell Sci* 2905–2916

Carsberg CJ, Myers KA and Stern PL (1996) Metastasis-associated 5T4 antigen disrupts cell-cell contacts and induces cellular motility in epithelial cells. *Int J Cancer* **27**: 84–92

Dall'Acqua W and Carter P (1998) Antibody engineering, *Curr Opin Struct Biol* **8**: 443–450

Dohlsten M, Hedlund G, Sjögren HO and Carlsson R (1998) Two subsets of human CD4+ T helper cells differing in kinetics and capacities to produce interleukin 2 and interferon-gamma can be defined by the Leu-18 and UCHL1 monoclonal antibodies. *Eur J Immunol* 1173–1178

Dohlsten M, Hedlund G, Åkerblom E, Lando P and Kalland T (1991) Monoclonal antibody-targeted superantigens: a different class of anti-tumour agents. *Proc Natl Acad Sci USA* **88**: 9287–9281

Dohlsten M, Bjorklund M, Sundstedt A, Hedlund G, Samson D, Kalland T (1993) Immunopharmacology of the superantigen Staphylococcal enterotoxin A in T-cell receptor Vb3 transgenic mice. *Immunology* **79**: 520–527

Dohlsten M, Abrahmsén L, Björk P, Lando PA, Hedlund G, Forsberg G, Brodin T, Gascoigne NRJ, Förberg C, Lind P and Kalland T (1994) Monoclonal antibody-superantigen fusion proteins: Tumour specific agents for T cell based tumour therapy. *Proc Natl Acad Sci* **91**: 8945–8949

Dohlsten M, Hansson J, Ohlsson L, Litton M and Kalland T (1995a) Antibody targeted superantigens are potent inducers of tumour-infiltrating T lymphocytes in vivo. *Proc Natl Acad Sci USA* **92**: 9791–9795

Dohlsten M, Lando PA, Björk P, Abrahmsén L, Ohlsson L, Lind P and Kalland T (1995b) Immuno-therapy of human colon cancer by antibody targeted superantigens. *Cancer Immunol. Immunotherapy* **41**: 162–168

Forsberg G, Forsgren M, Jaki M, Norin M, Sterky C, Enhörning Å, Larsson K, Ericsson M and Björk P (1997) Identification of framework residues in a secreted recombinant antibody fragment that control production level and localization in Escherichia coli. *J Biol Chem* **272**: 12430–12436

Garrido F, Ruiz-Cabello F, Cabrera T, Perez-Villar JJ, Lopez-Botet M, Duggan-Keen M and Stern PL (1997) Implications for immunosurveillance of altered HLA class I phenotypes in human tumours. *Immunol Today* **18**: 89–95

Goldenberg MM (1999) Traztusumab, a recombinant DNA-derived humanized monoclonal antibody, a novel agent for the treatment of metastatic breast cancer. *Clin Ther* **21**: 309–318

Hansson J, Ohlsson L, Persson R, Andersson G, Ilbäck N-G, Liton M, Kalland T and Dohlsten M (1997) Genetically engineered superantigens as tolerable antitumor agents. *Proc Natl Acad Sci USA* **94**: 2489–2494

Hedlund G, Dohlsten M, Lando PA and Kalland T (1990) Staphylococcal enterotoxins direct and trigger CTL killing of autologous HLA-DR+ mononuclear leukocytes and freshly prepared leukemia cells. *Cell Immunol* **129**: 426–434

Hole N and Stern PL (1988) A 72 KD trophoblast glycoprotein defined by a monoclonal antibody. *Br J Cancer* **57**: 239–246

Hole N and Stern PL (1990) Isolation and characterization of 5T4, a tumour-associated antigen. *Int J Cancer* **15**: 179–184

Hudson KR, Tiedemann RE, Urban RG, Lowe SC, Strominger JL and Fraser JD (1995) Staphylococcal enterotoxin A has two cooperative binding sites on major histocompatibility complex class II. *J Exp Med* **182**: 711–720

Hudson PJ (1998) Recombinant antibody fragments. *Curr Opin Biotechnol* **9**: 395–402

Hudson PJ (1999) Recombinant antibody constructs in cancer therapy. *Curr Opin Immunol* **11**: 548–557

Johnson HM, Russell JK and Pontzer CH (1991) Staphylococcal enterotoxin microbial superantigens. *FASEB J* **5**: 2706–2712

Litton MJ, Dohlsten M, Lando PA, Kalland T, Ohlsson L, Andersson J and Andersson U (1996) Antibody-targeted superantigen therapy induces tumour-infiltrating lymphocytes, excessive cytokine production, and apoptosis in human colon carcinoma. *Eur J Immunol* **26**: 1–9

Massague J (1987) *Methods Enzymol* **146**: 103–

McLaughlin P, Grillo-Lopez AJ, Link BK, Levy R, Czuczman MS, Williams ME, Heyman MR, Bence-Bruckler I, White CA, Cabanillas F, Jain V, Ho AD, Lister J, Wey K, Shen D and Dallaire BK (1998) Rituximab chimeric anti-CD20 monoclonal antibody therapy for relapsed indolent lymphoma: half of patients respond to a four-dose treatment program. *J Clin Oncol* **16**: 2825–2833

Modjtahedi H, Hickish T, Nicolson M, Moore J, Styles J, Eccles S, Jackson E, Salter J, Sloane J, Spencer L, Priest K, Smith I, Dean C, Gore M (1996) Phase I trial and tumour localisation of the anti-EGFR monoclonal antibody ICR62 in head and neck or lung cancer. *Br J Cancer* **73**: 228–235

Mulder WM, Stern PL, Stukart MJ, de Windt E, Butzelaar RM, Meijer S, Ader HJ, Claessen AM, Vermorken JB, Meijer CJ, Wagstaff J, Scheper RJ and Bloemena E (1997) Low intercellular adhesion molecule 1 and high 5T4 expression on tumor cells correlate with reduced disease-free survival in colorectal carcinoma patients. *Clin Cancer Res* **3**: 1923–1930

Myers KA, Rahi-Saund V, Davison MD, Young JA, Cheater AJ and Stern PL (1994) Isolation of a cDNA encoding 5T4 oncofetal trophoblast glycoprotein. An antigen associated with metastasis contains leucine-rich repeats. *J Biol Chem* **269**: 9319–9324

Ortegel JW, Staren ED, Faber LP, Warren WH and Braun DP (2000) Cytokine biosynthesis by tumor infiltrating T lymphocytes from human non small-cell lung carcinoma. *Cancer Immunol Immunother* **48**: 627–634

Riethmüller G, Schneider-Gaedicke E and Johnson JP (1993) Monoclonal antibodies in cancer therapy. *Curr Opin Immunol* **5**: 732–739

Riethmuller G, Holz E, Schlimok G, Schmiegel W, Raab R, Hoffken K, Gruber R, Funke I, Pichlmaier H, Hirche H, Buggisch P, Witte J and Pichlmayr R (1998) Monoclonal antibody therapy for resected Dukes' C colorectal cancer: seven-year outcome of a multicenter randomized trial. *J Clin Oncol* **16**: 1788–1794

Rosendahl A, Kristensson K, Carlsson M, Skartved NJ, Riesbeck K, Soegaard M and Dohlsten M (1999) Long-term survival and complete cures of B16-melanoma carrying animals after therapy with tumour targeted IL-2 and SEA. *Int J Cancer* **81**: 151–163

Scatchard G (1949) The attraction of proteins for small molecules and ions. *Ann NY Acad Sci* **51**: 660–672

Schad EM, Zaitseva I, Zaitsev VN, Dohlsten M, Kalland T, Schlievert DH, Ohlendorf DH and Svensson LA (1995) Crystal structure of the superantigen staphylococcal enterotoxin type A, *EMBO J* **14**: 3292–3301

Soegaard M, Ohlsson L, Kristensson K, Rosendahl A, Sjöberg A, Forsberg G, Kalland T and Dohlsten M (1999) Treatment with tumour-reactive Fab-IL-2 and Fab-Staphylococcal enteroxin A fusion proteins leads to sustained T cell activation and long-term survival of mice with established tumours. *Int J Oncol* **15**: 873–882

Southall PJ, Boxer GM, Bagshawe KD, Hole N, Bromley M and Stern PL (1990) Immunohistological distribution of 5T4 antigen in normal and malignant tissues. *Br J Cancer* **61**: 89–95

Starzynska T, Rahi V and Stern PL (1992) The expression of 5T4 antigen in colorectal and gastric carcinoma. *Br J Cancer* **66**: 867–869

Starzynska T, Marsh PJ, Schofield PF, Roberts SA, Myers KA and Stern PL (1994) Prognostic significance of 5T4 oncofetal antigen expression in colorectal carcinoma. *Br J Cancer* **69**: 899–902

Starzynska T, Wiechowska-Kozlowska A, Marlicz K, Bromley M, Roberts SA, Lawniczak M, Kolodziej B, Zyluk A and Stern PL (1998) 5T4 oncofetal antigen in gastric carcinoma and its clinical significance. *Eur J Gastroenterol Hepatol* **10**: 479–484

Van De Griend RJ, Girhart MJ, Van Krimpen BA and Bolhuis RLH (1984) Human T cell clones exerting multiple cytolytic activities show heterogeneity in susceptibility to inhibition by monoclonal antibodies. *J Immunol* **133**: 1222–1229

van de Loosdrecht AA, Nennie E, Ossenkoppele GJ, Beelen RH and Langenhuijsen MM (1991) Cell mediated cytotoxicity against U 937 cells by human monocytes and macrophages in a modified colorimetric MTT assay. A methodological study. *J Immunol Methods* **141**: 15–22

Vater CA, Reid K, Bartle LM, Goldmacher VS (1995) Characterization of antibody binding to cell surface antigens using plasma membrane-bound plate assay. *Anal Biochem* **224**: 39–50

Winter G and Milstein C (1991) Man-made antibodies. *Nature* **349**: 293–299

Zimmermann S, Wels W, Froesch BA, Gerstmayer B, Stahel RA and Zangemeister-Wittke U 1997 A novel immunotoxin recognising the epithelial glycoprotein-2 has potent antitumoural activity on chemotherapy-resistant lung cancer. *Cancer Immunol Immunother* **44**: 1–9

© 2001 Cancer Research Campaign
